# Environmentally Specific Transformational Leadership and Employee’s Pro-environmental Behavior: The Mediating Roles of Environmental Passion and Autonomous Motivation

**DOI:** 10.3389/fpsyg.2020.01408

**Published:** 2020-06-25

**Authors:** Zongbo Li, Jiaxin Xue, Rui Li, Hong Chen, Tingting Wang

**Affiliations:** ^1^School of Economics and Management, China University of Mining and Technology, Xuzhou, China; ^2^School of Labor and Human Resources, Renmin University of China, Beijing, China; ^3^School of Business, Soochow University, Suzhou, China; ^4^Institute of Education, Tsinghua University, Beijing, China

**Keywords:** environmentally specific transformational leadership, environmental passion, autonomous motivation, pro-environmental behaviors, multiple mediating effect

## Abstract

Organizational management practices in promoting sustainable development of the ecological environment are becoming a crucial way for enterprises to gain competitive advantages. However, whether the goal of such practices can be achieved depends on employees’ perception of environmental problems and the way they act. Therefore, it is important to stimulate employees’ pro-environment behaviors through management activities. Building on affective events theory and self-determination theory, we examined the effect of environmentally specific transformational leadership on employees’ pro-environmental behaviors (PEBs), as well as the potential mediating effects of environmental passion and autonomous motivation. A cross-sectional survey was conducted among 214 employees in China. Structural equation modeling was performed to test the theory-driven models. The results showed that environmentally specific transformational leadership positively predicted employees’ PEBs, and that environmental passion and autonomous motivation mediated this relationship, respectively. Furthermore, multiple-mediating testing results showed that environmental passion and autonomous motivation played sequential mediating roles in the link of environmentally specific transformational leadership to PEBs. This research unveiled environmental passion and autonomous motivation as underlying mechanisms that accounted for the link between transformational leadership and PEBs.

## Introduction

In recent years, as the ecological environment becomes increasingly worse, more and more enterprises begin to pay attention to and participate in the management practices of environmental problems (Ahmad, 2015). The management practices in promoting environmental sustainability, such as energy conservation, emission reduction, green innovation, process reengineering, and the adoption of environmental management systems, are becoming an important means for enterprises to gain competitive advantages (Molina-Azorín et al., 2015; Liu et al., 2016). The effectiveness of enterprises’ environmental management practices depends on employees’ perception and following behavior for environmental problems (Boiral, 2009). The pro-environment behaviors (PEBs) in the workplace are considered to have a promoting effect on enterprise environmental performance (Robertson and Barling, 2013). When employees realize the seriousness and importance of environmental problems and therefore perform corresponding environmental protection actions, the intuitive benefit is to reduce the waste of resources and save operating costs, and the ultimate benefit is to improve the organization’s environmental performance and obtain competitive advantages (Del Brío et al., 2007; Boiral et al., 2015). Given these widespread effects, it is hardly surprising that many researchers have recognized and called for empirical research to foster employees’ workplace PEBs within organizations (Norton et al., 2017; Robertson and Carleton, 2018). Unfortunately, there is a lack of research on this issue in the Chinese context where environmental issues are particularly salient to organizations today.

Leaders in an organization not only influence several traditional organizational outcomes, such as employee attitudes and behaviors, as well as organizational task, financial, and safety performance (Barling et al., 2002; Hannah et al., 2008), but also influence some emerging outcomes, such as the environmental performance. The leadership styles which leaders typically exhibit toward environment have been shown to be effective motivating employees’ PEBs (Graves et al., 2013; Robertson and Barling, 2013; Afsar et al., 2016; Raineri and Paillé, 2016; Robertson and Carleton, 2018). Among the various leadership styles, transformational leadership has received great attention and widespread recognition in the field of organizational management and has been found can effectively predict various employee behaviors (Judge and Piccolo, 2004; Nohe and Hertel, 2017). Traditionally, scholars have examined the effects of transformational leadership behaviors across different contexts. However, in recent years they have shifted to a focus in which the behaviors are tailored to predict a specific target, such as occupational safety (Barling et al., 2002) and environmental behaviors (Robertson and Barling, 2013). Following the research paradigm of target-specific transformational leadership in predicting specific outcomes, the present study attempts to explore how environmentally specific transformational leadership can facilitate employees’ PEBs.

Previous research has identified some mediators which may link environmentally specific transformational leadership with employees’ environmental behaviors, such as environmental passion (Robertson and Barling, 2013), autonomous and external motivation (Graves et al., 2013; Graves and Sarkis, 2018), environmental concern (Kura, 2016), perceived pro-environmental climate of coworkers (Robertson and Carleton, 2018), value congruence (Wang et al., 2018), and environmental belief (Kim et al., 2020). Although researchers have done a lot of foundational work on the internal mechanism of environment-oriented transformational leadership predicting employees’ PEBs, future theorizing and research are required to better understand the integrated mechanisms that facilitate PEBs, and, ultimately, provide guidance for organizational practice. In addition, the PEB in the workplace (e.g., double-sided printing) belongs to extra-role spontaneous behavior, which is not included in the scope of job responsibilities (Daily et al., 2009). According to previous literature, the occurrence of PEB mainly depends on specific inducing situations and the intrinsic motivation of individuals (Zerbe et al., 2008; Graves et al., 2013). Toward that end, drawing on the affective events theory (Weiss and Cropanzano, 1996) and self-determination theory (Deci and Ryan, 2008), this study proposes and tests an integrated model of the mechanisms that underlie employees’ PEBs. Specifically, both harmonious environmental passion and autonomous environmental motivation are introduced as mediators to investigate how environmentally specific transformational leadership predicts employees’ PEBs. Moreover, environmental transformational leaders are proposed to sequentially stimulate employees’ environmental emotional experience and intrinsic environmental motivation, which in turn promote employees to engage in pro-environmental activities. The research will be advanced by testing the indirect-effect sizes of two mediating variables and the multiple mediating mechanisms that link the relationship between leadership styles and PEBs.

## Theoretical Background and Hypotheses

### Environmentally Specific Transformational Leadership and Employee PEBs

Broadly speaking, PEBs are defined as the sustainably developing and using behaviors that people performed on the natural environment, or the behaviors that they tried to reduce the negative impact of their activities on the natural environment (Bissing-Olson et al., 2013). Narrowly speaking, PEBs are specific to the context of organizational management, referring to the autonomous environmentally friendly behaviors of employees in the workplace, such as actively recycling paper, saving water and electricity, etc. (Robertson and Barling, 2013; Norton et al., 2017). To sum up, workplace PEB is a proactive behavior toward environmental protection at the individual level, and employees have the freedom to choose whether to implement such behavior. According to previous research literature, leadership style in an organization is one of the key antecedents of employee PEB (Afsar et al., 2016; Kura, 2016; Robertson and Carleton, 2018; Wang et al., 2018; Tuan,2019a, b)

Transformational leadership is a positive and active leadership style comprised of four related behaviors: idealized influence, inspirational motivation, intellectual stimulation and individualized consideration (Bass, 1999; Judge and Piccolo, 2004; Schmitt et al., 2016). Although traditional transformational leadership has been widely recognized, more and more scholars have gradually realized the importance of transformational leadership tailored to predict a specific target since 2000. Barling et al. (2002) were the first to recognize the importance of target-specific transformational leadership in predicting specific outcomes. Specifically, they extended transformational leadership to occupational health and safety and conceptualized safety-specific transformational leadership. Subsequently, Beauchamp et al. (2010) and Morton et al. (2011) applied transformational leadership theory to classroom teaching and parenting behaviors, respectively. Based on this research, Robertson and Barling (2013) extended the focus of target-specific transformational leadership further by applying it to the environmental context. Environmentally specific transformational leadership is the specific management practice that transformational leadership focuses on environmental issues, and its contents are designed to encourage the environmental action of organizations or employees (Graves et al., 2013; Robertson and Barling, 2013; Robertson, 2018).

Different from traditional task-oriented leadership, environmentally specific transformational leadership focuses on the long-term sustainable development of the organization or society. It aims to promote the integration of individual environmental values and organizational environmental values, and to internalize organizational tasks into individual self-driven environmental behaviors (Robertson, 2018). Therefore, environmentally specific transformational leadership is more likely to motivate employees exhibiting environmental citizenship behaviors outside the job requirements. Similar to traditional transformational leadership, environmentally specific transformational leadership can be divided into four main behavior styles, each of which can be used to motivate PEBs of employees within the organization (Robertson and Barling, 2013; Robertson, 2018). Firstly, environmentally specific transformational leaders who show idealized influence behavior act as role models of employees by demonstrating environmental sustainability ideas, making commitments to followers, telling employees what is right, and encouraging subordinates to take environmentally friendly actions that benefit the natural environment (Robertson and Barling, 2013). When leaders exhibit these behaviors, employees are more likely to follow them and engage in PEBs. Secondly, environmentally specific transformational leaders high in inspirational motivation encourage employees to go beyond their individual needs for the collective interests (e.g., walk/bike/take the bus to work instead of driving) (Graves et al., 2013). They also inspire employees to overcome psychological setbacks and external obstacles through their passion and optimism (e.g., enrich environmental knowledge and participate in environmental activities), in order to transcend self-interest to engage in behaviors that can benefit the natural environment (Robertson and Barling, 2013; Robertson, 2018). Simultaneously, transformational leaders high in intellectual stimulation also encourage employees to think creatively about environmental issues and explore innovative solutions to environmental problems (Schmitt et al., 2016). In this context, environmental leadership behavior can encourage employees’ initiative for environmental protection and promote the emergence of employees’ PEBs, especially the environmental innovation behavior (Robertson and Carleton, 2018). Finally, environmentally specific transformational leaders can often establish a closer relationship with their followers by exhibiting individualized consideration, and thus transmit their environmental values to employees as well as inspire and shape their followers’ PEBs (Graves et al., 2013; Kura, 2016). Therefore, this study proposes:

Hypothesis 1: Environmentally specific transformational leadership positively predict employees’ PEBs.

### Mediating Role of Environmental Passion

Work passion is a psychological state characterized by experiencing strong positive emotions and recognizing the intrinsic driving force of work and the meaningful connection between individuals and work (Vallerand et al., 2003; Perttula and Cardon, 2011; Ho et al., 2018). Accordingly, environmental passion is defined as the strong emotional experience of employees toward environmentally friendly activities in the workplace. A person who is passionate about environmental protection not only practices environmentally friendly behaviors, but also calls himself/herself an environmentalist (Afsar et al., 2016). According to previous research, work passion includes harmonious work passion and obsessive work passion (Vallerand et al., 2003; Liu et al., 2011). Considering the spontaneity of PEB (i.e., the optional extra-role behavior), only the role of harmonious work passion for environment will be discussed when exploring the connection of environmentally specific transformational leadership and employees’ PEBs. In environmental activities, employees with harmonious environmental passion devote to the environmental protection activities because of their preference, rather than results-oriented incentives or external pressures (Afsar et al., 2016).

Drawing on the affective events theory (Weiss and Cropanzano, 1996), in the process of direct interaction between immediate leaders and subordinates, transformational leadership behaviors (including verbal or non-verbal behaviors) focusing on environmental issues can be regarded as specific affective events, which are crucial to evoke subordinates’ harmonious environmental passion (Liu et al., 2011; Robertson and Barling, 2013). Firstly, environmentally specific transformational leaders convey the determination and confidence of the organization to employees through their demonstration in environmental protection, potentially arousing the positive emotional expectations of environmental activities among employees (Walumbwa et al., 2008). Secondly, environmentally specific transformational leaders high in inspirational motivation and intellectual stimulation also encourage employees to solve environmental problems innovatively and guide employees to transcend their self-interests for the sake of organizational social responsibility and environmental sustainability, thus enhancing employees’ intrinsic force to engage in environmental protection activities (Xie and Zhang, 2012). Specifically, inspirational motivation will create optimism when individual contribution leads to the organizational environmental sustainability, and ignite employees’ passion. Thirdly, environmentally specific transformational leaders who exhibit individualized consideration (e.g., caring, mentoring) for employees should also make employees perceive the affective and instrumental support, so as to arouse the passion of employees who are more amenable to leaders’ guidance about environmental issues to engage in environmental protection activities (Judge and Piccolo, 2004). If employees have little opportunity to observe leaders exhibit or be encouraged in environmentally friendly behaviors, they are much less likely to exude work passion for environmental issues.

As a positive emotional experience, harmonious environmental passion will further promote employees’ PEBs in the workplace. First of all, the harmonious environmental passion experience has an incentive effect on behaviors, which can motivate individuals to engage in activities for achieving challenging goals (Vallerand et al., 2003). Corresponding to the harmonious work passion for environment, these kinds of activities mainly refer to the environmentally friendly behaviors involved in improving environmental problems (Astakhova, 2015). Secondly, positive emotional experience (e.g., happiness and excitement) is energetic and leaves individuals inspired to make a difference, which will result in a motivation to engage in environmentally friendly activity with passionate. Harmonious environmental passion is one kind of positive emotional experience (Perrewé et al., 2013). Finally, previous studies have found that harmonious work passion plays an important role in the mechanism by which management activities affect employee behaviors (Liu et al., 2011). Therefore, this study proposes:

Hypothesis 2: Harmonious environmental passion mediates the relationship between environmentally specific transformational leadership and employees’ PEBs.

### Mediating Role of Autonomous Motivation

According to self-determination theory, motivation is one of the important determinants of individual behavior, which can be divided into two categories − autonomous motivation and controlled motivation (Gagné and Deci, 2005; Deci and Ryan, 2008). Autonomous motivation is acknowledged as eliciting the behaviors an individual considers to be interesting, agreeable, or consistent with his/her intrinsic values or goals, while the behaviors induced by the controlled motivation are due to external or internal pressure (e.g., financial reward or punishment) (Pelletier et al., 2010; Graves et al., 2013). Considered the spontaneity of employees’ PEBs, environmentally specific transformational leadership is posited to inspire the employees’ autonomous motivation toward environmentally friendly behaviors.

As mentioned above, environmentally specific transformational leaders will enthusiastically talk about the importance of sustainable development of the environment, illustrate the environmental objectives of the organization, inspire employees to solve environmental problems innovatively, and emphasize the value of environmental management within a great vision (e.g., the ecological protection contributes to contemporary times and brings benefits for future centuries), which will facilitate employees’ internalization of the organization’s environmental values and enable employees to have a high self-actualization experience when practicing PEBs (Walumbwa et al., 2008; Daily et al., 2009). The importance of such environmental values in their self-identity construction will be enhanced as employees accept and internalize the values conveyed by leaders, thereby making it more meaningful to engage in the environmental protection activities (Turaga et al., 2010; Wesselink et al., 2017). Furthermore, the pro-environment action of transformational leaders toward environmental protection sets a good model for employees by which they can further internalize the values and goals of environmental sustainable development, thus enhancing their autonomous motivation for PEBs (Boiral et al., 2015; Yuriev et al., 2018).

Autonomous motivation for environmental protection will be further transformed into specific PEBs. Because of the spontaneity of the PEB, the autonomous motivation of employees to engage in environmental protection is consistent with the subsequent environmentally friendly behavior (Lu et al., 2017). According to self-determination theory, employees high in autonomous motivation will actively engage in corresponding environmental protection activities, even without external incentives (Gagné and Deci, 2005). Previous studies using student samples have shown that autonomous motivation can predict PEBs such as recycling, energy-saving, and green purchasing behavior (Osbaldiston and Sheldon, 2003). Even though some PEBs involve in creativity, such as green product design which requires individuals to solve complex problems innovatively, the importance of autonomous motivation in promoting green innovation behaviors has been identified (Gagné and Deci, 2005; Norton et al., 2015). Therefore, this study proposes:

Hypothesis 3: Autonomous environmental motivation mediates the relationship between environmentally specific transformational leadership and employees’ PEBs.

### Sequential Mediating Effects of Environmental Passion and Autonomous Motivation

Based on the above elaboration, environmentally specific transformational leadership can promote employees’ PEBs not only through environmental passion, but also through autonomous environmental motivation (Steg et al., 2014). The current study further proposes that both environmental passion and autonomous environmental motivation may exert sequential mediating effects on the relationship between environmentally specific transformational leadership and employees’ PEBs. That is, the arousal of environmental passion can, in turn, improve employees’ level of autonomous environmental motivation (Afsar et al., 2016). On the one hand, employees’ work passion for environmental protection is often associated with individuals’ positive emotional experiences. When being in a positive emotional state, employees have a stronger level of voluntary motivation to achieve their own goals, including environmental practice goals (Biraglia and Kadile, 2017). On the other hand, employees with harmonious environmental passion can perceive the autonomy in environmentally friendly behaviors, which may increase individuals’ interest in PEBs, and thus promote intrinsic motivation (Biraglia and Kadile, 2017). In short, if employees highly identify with and show strong passion in the significance of PEBs, they will have a stronger autonomous motivation to participate in such environmentally friendly behaviors. Therefore, this study proposes:

Hypothesis 4: Harmonious environmental passion and autonomous environmental motivation sequentially mediate the relationship between environmentally specific transformational leadership and employees’ PEBs.

**FIGURE 1:**
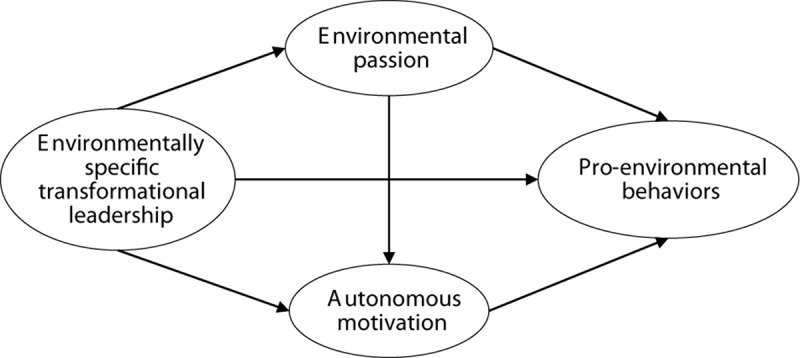
Hypothetical model.

The hypothetical model this study proposes in the present study is depicted in Figure 1.

## Materials and Methods

### Participants and Procedures

#### Participants

The participants in this study consisted of two parts. The main part of participants in this study were full-time employees from two manufacturing companies in a major city located in central China. The production activities of each targeted companies had a huge impact on the local environment quality (e.g., water pollution, air pollution, and solid waste pollution, etc.), so that employees are sensitive to environmental protection issues. In this survey, the data were collected via paper-and-pencil questionnaires. In total, 190 questionnaires were issued, and 178 were returned for a response rate of 93.7%. Questionnaires less than 70% completed and those that contained illegible or regular responses were removed (22 responses). Following these exclusions, 156 valid questionnaires remained for a valid response rate of 82.1%.

In order to strengthen the explaining power and the generalizability of our findings, the current study expanded the research sample to employees from other companies in multiple industries. These participants were invited to fill in an electronic questionnaire through the link of the web-based survey. In total, 63 electronic questionnaires were sent and 58 valid questionnaires were received, resulting in a response rate of 92.1%.

The independent sample *T*-test was employed to analyze the difference of sample data collected in two ways (Podsakoff et al., 2012). The results showed that two sample data had no significant difference and could be synthesized into a single data set for the following analyses. The current study collected 214 valid employee questionnaires from two ways totally with a valid response rate of 84.6%. Among the final sample, 44.4% of the valid respondents were male. The average age was 28.4 years (*SD* = 5.59). The majority of the respondents had college experience (46.7%) and vocational school diplomas (32.7%). In terms of job tenure, 28.5% of the employees have been with the company for 2−24 months, 41.1% for 2 to 5 years, 19.2% from 5 to 10 years and 11.2% for over 10 years. In addition, 14.5% of the participants had a low or mid-level leadership position.

#### Procedures

In the field survey, researchers first contacted the human resource director of each company and then asked whether their companies are willing to participate in this survey. After getting approval, the director of each company introduced an inside research helper within the company for this survey. Inside helpers from two companies are human resource department staff who are equipped with job experience in personnel assessment. After being selected, the inside helpers were briefed on the purpose of this study, the proper way of collecting data, and the matters requiring attention. In addition, two student research assistants were arranged to enter the two companies successively, helping distribute and collect questionnaires together. The first author of this paper also took part in the survey process as he offered guidance and assistance for the student assistants and inside helpers.

Assisted by the research assistants, 190 questionnaires were sent to employees at the two companies. Before the questionnaires were filled out, all participants were informed that what is the purpose of the study, that participation was voluntary, and that participants’ privacy would be strictly protected. Invitees were required to complete the paper-and-pencil questionnaire within 10 min in a designated meeting room. After completing the questionnaire, respondents sealed the completed questionnaire in an envelope for confidentiality and returned it directly to the research assistant. To convey our appreciation, participants were offered a high-quality pen as a gift of completing the questionnaire.

In the web-based survey, announcements were posted on social media first to recruit participants who were employed by the company and had an immediate supervisor. The intention of this study and the voluntariness of participation in the survey were also explained in the announcements. In the limited week, we recruited a total of 63 employees to participate in this survey voluntarily. All participants were sent an online link of the questionnaire and required to complete it within 8 h. To show our appreciation, each participant completed the questionnaire was offered 15 CNY through electronic payment.

This study has been conducted in accordance with the recommendations of the Science & Technology Research Office of CUMT. There were no unethical behaviors during the research process, and this study was exempt from further ethics board approval since our study did not involve human clinical trials or animal experiments.

### Measurement

The survey conducted in the present study were originally in English. The English version instrument were translated into the Chinese version. To ensure the reliability and validity of the scales, two Chinese bilingual professional translators were asked to complete the translation-back translation procedures independently with the guidance of the double blinded principle (Brislin, 1986), which had been widely used in studies of non-English speaking countries (Aryee et al., 2007).

#### Environmentally Specific Transformational Leadership

A five-item shortened version of the Environmental Transformational Leadership Scale introduced by Graves et al. (2013) was employed to measure employees’ perceptions of the immediate supervisor’s environmentally specific transformational leadership. This scale was modified from the Multifactor Leadership Questionnaire (MLQ - 5x) developed by Bass and Avolio (1995), evaluating five aspects of transformational leadership, respectively, namely idealized influence - attributes, idealized influence - behaviors, inspirational motivation, intellectual stimulation and individualized consideration. Response options ranged from 1 (*never*) to 5 (*always*). Sample items are “My supervisor talks about the importance of protecting nature,” and “My supervisor provides teaching and coaching on environmental issues.” In the present study, the scale’s alpha reliability was 0.93.

#### Environmental Passion

An eight-item shortened version of the Harmonious Environmental Passion Scale developed by Robertson and Barling (2013) was used to measure employees’ harmonious passion for the environment. Two items were deleted from the original ten items because they were not mainly generated in the workplace and did not reflect the actual situation of Chinese enterprises, which were “I am a volunteered member of an environmental group” and “I have voluntarily donated time or money to help the environment in some way.” Response options ranged from 1 (*strongly disagree*) to 5 (*strongly agree*). Sample items include “I am passionate about the environment,” and “I enjoy engaging in environmentally friendly behaviors.” In the present study, the scale’s alpha reliability was 0.87.

#### Autonomous Environmental Motivation

A six-item Autonomous Motivation Scale developed by Graves et al. (2013) was used to measure employees’ autonomous motivation (three items each for the identified and intrinsic motivation) to engage in PEBs at work. Consistent with the study of Graves et al. (2013), confirmatory factor analysis (CFA) indicated that the six items represented a single factor, rather than two separate factors (not reported here). Response options ranged from 1 (*strongly disagree*) to 5 (*strongly agree*). Sample items are “It allows me to achieve goals I consider important (identified motivation),” and “Of the pleasure I get from doing it (intrinsic motivation).” In the present study, the scale’s alpha reliability was 0.90.

#### Pro-environmental Behaviors

A seven-item Workplace Pro-environmental Behaviors Scale developed by Robertson and Barling (2013) was used to measure employees’ pro-environmental behaviors in the workplace, such as printing double-sided, conveniently turning unused electrical appliances off, and giving suggestions about environmental protection, etc. Response options ranged from 1 (*never*) to 5 (*always*). Sample items include “I put recyclable material (e.g., cans, paper, bottles, batteries) in the recycling bins,” and “I take part in environmentally friendly programs (e.g., bike/walk to work day, bring your own local lunch day).” In the present study, the scale’s alpha reliability was 0.90.

## Results

### Common Method Variance

Common method variance (CMV) can inflate relationships when the data are collected from a single source (Podsakoff et al., 2003). Harman’s single-factor test was applied to test whether the majority of the variance could be accounted for by one general factor (Podsakoff et al., 2003). The logic underlying the single-factor test is that if method variance is largely responsible for the covariation among the measures, factor analysis should find a single factor fitting the data. The results showed that the first factor accounted for only 27.35% of the variance, less than half of total variance (65.49%), which was acceptable according to the criteria suggested by previous researchers (Podsakoff and Organ, 1986; Fuller et al., 2016). Furthermore, CFA similarly showed that the fit of the single factor model was poor. Our hypothesized four-factor model was significantly better fit than the single-factor model (Williams et al., 2010).

Moreover, to further determine whether CMV is problematic in this study, the CFA marker technique was employed (Simmering et al., 2015). The CFA (five-factor) model was built by adding the CMV variable to the four-factor model. Compared with the four-factor model, the CFA five-factor model is no better. Further, the chi-square difference also did not reach the significant level [Δχ^2^(*df*) = 35.24(26), *p* > 0.05]. Taken together, it can be concluded that the CMV was negligible in this study (see Table 1).

**TABLE 1:** Comparison of measurement models.

Structure	χ ^2^	*df*	χ ^2^/*df*	RMSEA	CFI	NNFI	Δχ *2*(*df*)
Four-factor (baseline)	791.84	293	2.70	0.07	0.96	0.95	
One-factor	2483.37	299	8.31	0.19	0.87	0.85	1691.53(6)
Three-factor	1263.11	296	4.27	0.12	0.93	0.92	471.27(3)
Five-factor	756.60	267	2.83	0.07	0.96	0.95	35.24(26)

*RMSEA, root mean square error of approximation; CFI, comparative fit index; NNFI, non-normed fit index.*

### Measurement Model Testing

The CFA is conducted to test the construct distinctiveness of four major variables of environmentally specific transformational leadership, environmental passion, autonomous motivation, and PEBs (see Table 1). The hypothesized four-factor baseline model provided a good fit with all fit indices within acceptable levels. In addition to the baseline model, two alternative nested models were tested, i.e., three-factor model (two mediators were combined into one factor) and one-factor model (All four variables merged into a single factor). As shown in Table 1, the four-factor model fit the data better than the two alternative models by using the chi-square change statistic [Compared with three-factor model: Δχ^2^(*df*) = 471.27(3), *p* < 0.01; Compared with one-factor model: Δχ^2^(*df*) = 1691.54(6), *p* < 0.01] (Bentler and Bonett, 1980). In addition, the changes in the comparative fit index (CFI) between the four-factor model and the alternatives were greater than 0.02, suggesting a significant improvement in model fit (Cheung and Rensvold, 2002). The hypothesized four-factor model was, therefore, the most appropriate representation of the factor structure of the items.

### Descriptive Statistics and Correlations

The means and standard deviations of and the correlations between each of the variables are presented in Table 2. In line with previous research, environmentally specific transformational leadership, environmental passion, and autonomous motivation were all positively related to PEBs. Environmentally specific transformational leadership was also positively correlated with environmental passion and autonomous motivation. The correlation table offers a first insight into all hypothesized relationships among the concepts.

**TABLE 2:** Means, standard deviations and intercorrelations among variables.

Variable	*M*	*SD*	1	2	3	4
(1) Environmentally specific transformational leadership	3.34	0.99	−			
(2) Environmental passion	3.89	0.57	0.44**	−		
(3) Autonomous motivation	3.89	0.79	0.53**	0.58**	−	
(4) Pro-environmental behaviors	3.91	0.69	0.44**	0.61**	0.64**	−

*p < 0.05 and **p < 0.01.

### Structural Model Testing

Structural equation modeling (SEM) using maximum likelihood estimation in Mplus 8.3 was conducted to test our hypotheses and to assess the appropriateness and fit of our proposed theoretical model. First, a direct-effect model was built (Model 1) to test the relationship between environmentally specific transformational leadership and employees’ PEBs. The results showed that Model 1 fit the data well, χ^2^(53) = 204.60, *p* < 0.01; RMSEA = 0.08; CFI = 0.94; TLI = 0.92. The path from environmentally specific transformational leadership to employees’ PEBs was positively significant (β = 0.48, *p* < 0.01). Therefore, hypothesis 1 was supported.

**FIGURE 2:**
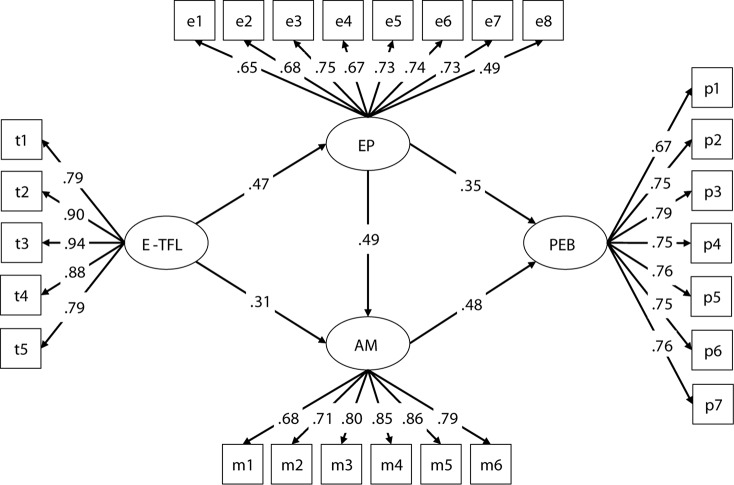
The ultimate mediation model (Model 1). Note: E-TFL, environmentally specific transformational leadership; EP, environmental passion; AM, autonomous motivation. PEB, pro-environmental behavior. All path coefficients and factor loading were significant at *p* < 0.01.

Second, the Hypothetical model was tested (partial multiple mediated model) (see Figure 1). The results indicated that the Hypothetical model fit well to the data (see Table 3), but the path from environmentally specific transformational leadership to employees’ PEBs was non-significant (β = 0.08, *p* > 0.05). Thus, this study built an alternative Model 1 (see Figure 2), in which the direct path from environmentally specific transformational leadership to employees’ PEBs was deleted. The results showed that Model 1 also fit the data well. Compared with the Hypothetical model, the chi-square difference did not reach the significant level [Δχ^2^(1) = 1.30, *p* > 0.05], suggesting that Model 1 did not fit the data better than the Hypothetical model. However, Model 1 was acceptable according to the simplicity principle of the structural equation model.

**TABLE 3:** Comparison of the structural models.

Model	χ ^2^	*df*	χ ^2^/*df*	RMSEA	CFI	TLI
Hypothetical model	780.45	293	2.66	0.07	0.96	0.93
**Model 1**	**781.75**	**294**	**2.66**	**0.07**	**0.96**	**0.93**
Model 2	828.31	294	2.82	0.08	0.95	0.92
Model 3	829.00	295	2.81	0.08	0.95	0.92

*Model 1 with bold values specified marked refers to the final model.*

To determine whether the two mediators are parallel or serial, and to find the most satisfactory model, another alternative model (Model 2) was developed then, in which a path from environmental passion to autonomous motivation was deleted from the Hypothetical model. The results demonstrated that Model 2 fit the data well (see Table 3), but the direct-path from environmentally specific transformational leadership to employees’ PEBs was still non-significant (β = 0.06, *p* > 0.05). Thus, the direct-path from model 2 was deleted and an alternative Model 3 was built. The results showed that Model 3 fit the data well as well. Through comparison of the chi-square change between Model 2 and the Hypothetical model [Δχ^2^(1) = 47.86, *p* < 0.01], Model 2 and Model 1 [Δχ^2^ = 45.56, Δ*df* = 0], Model 3 and the Hypothetical model [Δχ^2^(2) = 48.55, *p* < 0.01], and Model 3 and Model 1 [Δχ^2^(1) = 47.25, *p* < 0.01], the significant level was reached, revealing that Model 2 and Model 3 significantly deteriorate model fit (see Table 3). Taken together, Model 1 was selected as our study’s structural model (see Figure 2). Moreover, the factor loading of each indicator exceeded 0.65 except one indicator whose factor loading was 0.49, and all reached the significant level (*p* < 0.01) (see Figure 2).

Furthermore, the bootstrapping method was used to test the mediation effects in Model 1. The most accurate estimation of indirect effects can be obtained by bootstrap sampling. If zero is not included in the 95% confidence interval, indirect effects are significant. The SEM results showed that our hypotheses are all supported (see Figure 2 and Tables 3, 4). First, the total effect from environmentally specific transformational leadership to PEBs was significant, supporting hypothesis 1. Second, the simple indirect effect of environmentally specific transformational leadership on PEBs through environmental passion was significant, and the path coefficients from environmentally specific transformational leadership to environmental passion, and from environmental passion to PEBs were significant, supporting hypothesis 2. Third, the simple indirect effect of autonomous motivation in the link between environmentally specific transformational leadership and PEBs was significant, and the path coefficients from environmentally specific transformational leadership to autonomous motivation, and from autonomous motivation to PEBs were significant as well, supporting hypothesis 3. Fourth, the multiple indirect effect from environmentally specific transformational leadership to PEBs sequentially mediated by environmental passion and autonomous motivation was significant, and the path coefficient from environmental passion to autonomous motivation was also significant, supporting hypothesis 4.

**TABLE 4:** Direct and indirect effects and 95% confidence intervals in final model 1.

Model pathways	Estimated effect	95% CIs
**Total effect**		
E-TFL→PEBs	0.43**	[0.32, 0.53]
**Direct effects**		
E-TFL→EP	0.47**	[0.36, 0.57]
E-TFL→AM	0.31**	[0.20, 0.41]
EP→PEBs	0.35**	[0.15, 0.51]
AM→PEBs	0.48**	[0.32, 0.63]
EP→AM	0.49**	[0.37, 0.61]
**Indirect effects**		
P1: E-TFL→EP→PEBs	0.16**	[0.07, 0.26]
P2: E-TFL→AM→PEBs	0.15**	[0.08, 0.24]
P3: E-TFL→EP→AM→PEBs	0.11**	[0.07, 0.18]

The number of bootstrap samples for bias-corrected bootstrap confidence intervals is 5000. **p < 0.01.

The above results indicated that the process of environmental transformational leadership predicting employees’ PEBs relied on a multiple mediation model. Referring to Preacher and Hayes (2008), this study compared the three mediating pathways (i.e., P1, P2, and P3; see Table 4) using the multivariate delta method. The results showed that the difference between the two simple mediating pathways of environmental passion (i.e., P1) and autonomous motivation (i.e., P2) was 0.020, which did not reach a significant level (*Z* = 0.14, *p* > 0.05), suggesting that the two simple mediating pathways were not superior to each other. Similarly, this study compared the differences between multiple mediating pathway (i.e., P3) and two simple mediating pathways (i.e., P1 and P2). The results showed that there were no differences between P3 and P1 (*Z* = 1.48, *p* > 0.05), as well as between P3 and P2 (*Z* = 1.10, *p* > 0.05), suggesting that the multiple mediating pathway was not better than the two simple mediating pathways although it had statistical significance.

## Discussion

Drawing on affective events theory and self-determination theory, the present study integrates environmental passion and autonomous environmental motivation into one comprehensive framework, which systematically and uniquely tests the mechanisms linking environmentally specific transformational leadership to employees’ PEBs from the perspectives of emotional arousal and motivation stimulation. One of the contributions of this study is that the three indirect paths from environmentally specific transformational leadership to employees’ PEBs are compared. Another contribution of this study is that the sequential mediating pathways are identified, through which environmentally specific transformational leadership facilitates the employees’ PEBs. In what follows, the central findings are summarized and the contributions to the field of transformational leadership and PEBs are discussed.

Firstly, the positive prediction of environmentally specific transformational leadership to PEBs suggests that transformational leadership focusing on environmental issues is critical to employees’ environmentally friendly behavior in the workplace. Drawing on the theory of transformational leadership, transformational leadership behavior (e.g., describe organizational values and vision) has great inspiring attributes (Bass, 1999), which will be obvious especially when leaders exhibit the behavior aligning with the organization environmental value. This is because employees can learn the behavior pattern from leaders by close observation, which is helpful to promote the internalization of environmental protection value, and then stimulate employees to perform similar environmentally friendly behaviors. This standpoint has been confirmed in only a few previous studies (Graves et al., 2013; Robertson and Barling, 2013; Afsar et al., 2016; Tuan, 2019b). They found that both transformational leadership and spiritual leadership behavior with inspirational characteristics can better motivate employees’ environmentally friendly behaviors. In general, the current study suggests that leadership behavior aimed at addressing environmental issues can effectively facilitate employees’ PEBs in the workplace.

Secondly, harmonious work passion for the environment played as a mediator in the link of environmentally specific transformational leadership and employees’ PEBs. Previous studies have shown that employees’ immediate managers are the proximal agents of the organization and are likely to be critical in encouraging employees’ PEBs (Graves and Sarkis, 2018). Immediate leaders’ behaviors focusing on environmental issues, such as vision description, inspirational motivation, intellectual stimulation, individualized consideration, and support could be regarded as series of affective events, which would arouse employees’ positive emotional experience for the environment and further elicit their PEBs. This inference was consistent with the viewpoint of affective events theory, which argues that affective events can cause individual emotional experience, and then trigger a behavioral response (Weiss and Cropanzano, 1996). In previous studies, Robertson and Barling (2013) employed social learning theory to explain the mechanism of environmental passion in promoting individual PEBs. They posited that employees could learn by observing leaders’ pro-environmental behavior, be passionate about environmental activities and then engage in PEBs. However, there is a process of conscious awakening from the act of observation to the act of action, and alternative observation does not necessarily lead to explicit behavior. Therefore, only when employees internalize the environmental values can they perform more PEBs. The current research provides another important explanation framework for the relationship between environmentally specific transformational leadership and employees’ PEBs, and also expands the applicable scope of affective events theory in explaining organizational phenomena.

Thirdly, autonomous motivation also has a mediating effect on the relationship between environmentally specific transformational leadership and employees’ PEBs. After comparing the two simple mediating effects, the current study showed that there was no significant difference between the two indirect effects sizes, which indicated that compared with the emotional arousal path, the motivation stimulation path played the same role in linking leadership style with employees’ PEBs. The mediating role of autonomous motivation has also been confirmed in previous similar studies by Duan and Huang (2014) based on self-determination theory. Their study on voice behavior with the same characteristics of spontaneity found that internal motivation played an important role in explaining the process of transformational leadership in facilitating proactive behavior. Graves et al. (2013) also drew an analogous conclusion in their study, which is consistent with our study. However, they focused on the differential mediation between internal motivation and external motivation, as well as the situational conditions for internalization of external motivation. The current study not only focused on autonomous environmental motivation, but also focused on the differences between autonomous environmental motivation and harmonious environmental passion as mediators, which provided cross-perspective evidence to explain the relationship between transformational leadership style and employees’ PEBs.

Finally, this study integrated affective events theory and self-determination theory, and empirically verified the sequential mediating effects of environmental passion and autonomous motivation through which environmentally specific transformational leadership facilitated employees’ PEBs in the workplace. The evidence this study provided implied that the positive environmental emotional experience aroused by the environmentally specific transformational leadership could further promote the internalization of environmental motivation, which in turn motivate environmentally friendly behaviors. Although there is no evidence that this sequential mediating pathway of stimulating PEB is more effective than the simple mediating pathways, PEBs generated following emotional arousal and motivational stimulation is persistent. In summary, this study proposed and verified a more comprehensive theoretical framework, expanded the explanation scope of affective events theory and self-determination theory, and enriched research on employees’ green organizational behavior.

### Practical Implication

The proposed model highlights two ways that leaders might influence employees’ organizational greening activity, which has several practical implications for organizational management. First, environmentally specific transformational leadership was important for promoting employees’ PEBs. Considering the trainability of transformational leadership, it is suggested that organizations can incorporate green management into leadership development courses to help leaders improve their ability of solving environmental problems, resulting in guiding employees’ PEBs. Additionally, organizations can promote employees’ PEBs in two ways, namely, awakening environmental passion and internalizing environmental motivation. On the one hand, leaders can integrate the environmental value into the self-construction of individual work significance by describing the severity of environmental problems, in order to arouse employees’ positive emotional experience and cognition of environmental protection. On the other hand, leaders can activate employees’ autonomous environmental motivation by inspiring individuals to keep consistent with the organization’s environmental goals. Finally, from the perspective of employees, organizations should attach importance to the assessment of candidates’ environmental values in the recruitment and selection process. Individuals with high environmental values are more likely to be motivated to engage in PEB in the workplace than those with low environmental values.

### Limitations and Future Directions

Several aspects of this study warrant some caution and suggest additional avenues for future research. First, the cross-sectional nature of data used in the current study precludes any causal inferences. Future research should go beyond the constraints of the cross-sectional data and include longitudinal or time-lagged data to allow for causal inference. Second, although the results of *post hoc* statistical tests showed that there was no serious common method deviation in the current study, it was not completely excluded. Subsequent studies may collect data from multisource responses. Third, the current study only focused on the mediating mechanisms linking environmental transformational leadership with employees’ PEBs, ignoring its boundary conditions. According to the contingency theory of leadership, the effectiveness of leadership is constrained by specific situational conditions (Thompson and Vecchio, 2009), such as the environmental climate at the organizational level (Norton et al., 2012) and the supervisor’s organizational embodiment at the individual level (Eisenberger et al., 2010). Future research should incorporate these situational factors as moderators into the research framework. Fourth, only the intrinsic incentive mechanism of PEB was examined. Indeed, external pressure or incentive can also accelerate such behavior of individuals (Lavergne et al., 2010), such as compulsive institutional regulations or external rewards. Future research taking both internal factors and external factors into consideration are required when focusing on organizational incentive strategies development. Fifth, the ultimate goal of enterprise environmental management is not to motivate employees’ PEBs, but to improve the organization’s environmental performance and financial performance. Future research can add enterprise environmental performance, green innovation and even financial performance as outcomes into the research framework (Boiral, 2009), in order to expand and deepen the research on PEB.

## Conclusion

From the perspectives of emotional arousal and motivation stimulation, the present paper proposed and tested the mediating mechanisms of harmonious environmental passion and autonomous environmental motivation between environmentally specific transformational leadership and employees’ PEBs. There was no significant difference between the emotional arousal path and the motivation stimulation path of the environmentally specific transformational leadership in facilitating employees’ PEBs. In addition, organizational leaders could also promote the PEBs of employees through the path of emotional arousal and then motivation stimulation, which had been found to be effective here. By revealing the internal mechanisms, the organization could purposefully promote the PEBs of employees through the fostering of leadership, and then implemented various environmental management strategies.

## Data Availability Statement

The datasets analyzed in this article are not readily available due to the confidentiality agreement signed in advance between the ZL and the two manufacturing companies under investigation. This is also to protect respondent confidentiality and participant privacy. Requests to access the datasets should be directed to ZL, rancho_lee@163.com.

## Ethics Statement

The studies involving human participants were reviewed and approved by China University of Mining and Technology Ethics Committee. The patients/participants provided their written informed consent to participate in this study.

## Author Contributions

ZL, RL, and HC conceived and designed the framework. ZL, JX, and TW collected and analyzed the data. All authors contributed to writing the manuscript.

## Conflict of Interest

The authors declare that the research was conducted in the absence of any commercial or financial relationships that could be construed as a potential conflict of interest.

## Appendix: Survey Items

### Environmentally Specific Transformational Leadership

My supervisor:

1. Displays confidence about environmental issues (idealized influence attributes).
2. Talks about the importance of protecting nature (idealized influence behaviors).
3. Talks enthusiastically about what we need to do to protect nature (inspirational motivation).
4. Gets me to look at environmental problems in new ways (intellectual stimulation).
5. Provides teaching and coaching on environmental issues (individualized consideration).

### Environmental Passion

1. I am passionate about the environment.
2. I enjoy practicing environmentally friendly behaviors.
3. I enjoy engaging in environmentally friendly behaviors.
4. I take pride in helping the environment.
5. I enthusiastically discuss environmental issues with others.
6. I get pleasure from taking care of the environment.
7. I passionately encourage others to be more environmentally responsible.
8. I feel strongly about my environmental values.

### Autonomous Motivation

I would engage in green behaviors at work because:

1. It allows me to achieve goals I consider important (identified motivation).
2. It fits my own values (identified motivation).
3. It is personally important to me (identified motivation).
4. I enjoy it (intrinsic motivation).
5. Of the pleasure I get from doing it (intrinsic motivation).
6. It is fun (intrinsic motivation).

### Workplace Pro-environmental Behavior

1. I print double sided whenever possible.
2. I put compostable items in the compost bin.
3. I put recyclable material (e.g., cans, paper, bottles, and batteries) in the recycling bins.
4. I bring reusable eating utensils to work (e.g., travel coffee mug, water bottle, reusable containers, reusable cutlery).
5. I turn lights off when not in use.
6. I take part in environmentally friendly programs (e.g., bike/walk to work day, bring your own local lunch day).
7. I make suggestions about environmentally friendly practices to managers and/or environmental committees, in an effort to increase my organization’s environmental performance.

## References

[B1] Afsar B. Badir Y. Kiani U. S. (2016). Linking spiritual leadership and employee pro-environmental behavior: the influence of workplace spirituality, intrinsic motivation, and environmental passion. *J. Environ. Psychol.* 45 79–88. 10.1016/j.jenvp.2015.11.011

[B2] Ahmad S. (2015). Green human resource management: policies and practices. *Cogent Bus. Manag.* 2 1–13. 10.1080/23311975.2015.1030817

[B3] Aryee S. Chen Z. X. Sun L. Y. Debrah Y. A. (2007). Antecedents and outcomes of abusive supervision: test of a trickle-down model. *J. Appl. Psychol.* 92 191–201. 10.1037/0021-9010.92.1.191 17227160

[B4] Astakhova M. N. (2015). The curvilinear relationship between work passion and organizational citizenship behavior. *J. Bus. Ethics* 130 361–374. 10.1007/s10551-014-2233-5

[B5] Barling J. Loughlin C. Kelloway E. K. (2002). Development and test of a model linking safety-specific transformational leadership and occupational safety. *J. Appl. Psychol.* 87 488–496. 10.1037/0021-9010.87.3.488 12090606

[B6] Bass B. M. (1999). Two decades of research and development in transformational leadership. *Eur. J. Work Organ. Psychol.* 8 9–32. 10.1080/135943299398410

[B7] Bass B. M. Avolio B. (1995). *Multifactor Leadership Questionnaire.* Redwood City, CA: Mindgarden.

[B8] Beauchamp M. R. Barling J. Li Z. Morton K. L. Keith S. E. Zumbo B. D. (2010). Development and psychometric properties of the transformational teaching questionnaire. *J. Health Psychol.* 15 1123–1134. 10.1177/1359105310364175 20522503

[B9] Bentler P. M. Bonett D. G. (1980). Significance tests and goodness of fit in the analysis of covariance structures. *Psychol. Bull.* 88 588–606. 10.1037/0033-2909.88.3.588

[B10] Biraglia A. Kadile V. (2017). The role of entrepreneurial passion and creativity in developing entrepreneurial intentions: insights from american homebrewers. *J. Small Bus. Manag.* 55 170–188. 10.1111/jsbm.12242

[B11] Bissing-Olson M. J. Iyer A. Fielding K. S. Zacher H. (2013). Relationships between daily affect and pro-environmental behavior at work: the moderating role of pro-environmental attitude. *J. Organ. Behav.* 34 156–175. 10.1002/job.1788

[B12] Boiral O. (2009). Greening the corporation through organizational citizenship behaviors. *J. Bus. Ethics* 87 221–236. 10.1007/s10551-008-9881-2

[B13] Boiral O. Talbot D. Paillé P. (2015). Leading by example: a model of organizational citizenship behavior for the environment. *Bus. Strat. Environ.* 24 532–550. 10.1002/bse.1835

[B14] Brislin R. W. (1986). “The wording and translation of research instruments,” in *Field Methods in Cross-Cultural Research*, eds Lonner W. J. Berry J. W. (Thousand Oaks, CA: Sage Publications, Inc), 137–164.

[B16] Cheung G. W. Rensvold R. B. (2002). Evaluating goodness-of-fit indexes for testing measurement invariance. *Struct. Equ. Model.* 9 233–255. 10.1207/S15328007SEM0902_5

[B18] Daily B. F. Bishop J. W. Govindarajulu N. (2009). A conceptual model for organizational citizenship behavior directed toward the environment. *Bus. Soc.* 48 243–256. 10.1177/0007650308315439

[B19] Deci E. L. Ryan R. M. (2008). Self-determination theory: a macrotheory of human motivation, development, and health. *Can. Psychol.* 49 182–185. 10.1037/a0012801

[B20] Del Brío J. Á Fernández E. Junquera B. (2007). Management and employee involvement in achieving an environmental action-based competitive advantage: an empirical study. *Int. J. Hum. Resour. Manag.* 18 491–522. 10.1080/09585190601178687

[B21] Duan J. Huang C. (2014). The mechanism of individual-focused transformational leadership on employee voice behavior: a self-determination perspective. *Nankai Bus. Rev.* 17 98–109.

[B22] Eisenberger R. Karagonlar G. Stinglhamber F. Neves P. Becker T. E. Gonzalez-Morales M. G. (2010). Leader-member exchange and affective organizational commitment: the contribution of supervisor’s organizational embodiment. *J. Appl. Psychol.* 95 1085–1103. 10.1037/a0020858 20718516

[B23] Fuller C. M. Simmering M. J. Atinc G. Atinc Y. Babin B. J. (2016). Common methods variance detection in business research. *J. Bus. Res.* 69 3192–3198. 10.1016/j.jbusres.2015.12.008

[B24] Gagné M. Deci E. L. (2005). Self-determination theory and work motivation. *J. Organ. Behav.* 26 331–362. 10.2307/4093832

[B25] Graves L. M. Sarkis J. (2018). The role of employees’ leadership perceptions, values, and motivation in employees’ provenvironmental behaviors. *J. Clean. Product.* 196 576–587. 10.1016/j.jclepro.2018.06.013

[B26] Graves L. M. Sarkis J. Zhu Q. (2013). How transformational leadership and employee motivation combine to predict employee proenvironmental behaviors in China. *J. Environ. Psychol.* 35 81–91. 10.1016/j.jenvp.2013.05.002

[B27] Hannah S. T. Avolio B. J. Luthans F. Harms P. D. (2008). Leadership efficacy: review and future directions. *Leadership Q.* 19 669–692. 10.1016/j.leaqua.2008.09.007

[B28] Ho V. T. Kong D. T. Lee C. H. Dubreuil P. Forest J. (2018). Promoting harmonious work passion among unmotivated employees: a two-nation investigation of the compensatory function of cooperative psychological climate. *J. Vocat. Behav.* 106 112–125. 10.1016/j.jvb.2018.01.005

[B29] Judge T. A. Piccolo R. F. (2004). Transformational and transactional leadership: a meta-analytic test of their relative validity. *J. Appl. Psychol.* 89 755–768. 10.1037/0021-9010.89.5.755 15506858

[B30] Kim W. G. McGinley S. Choi H. M. Agmapisarn C. (2020). Hotels’ environmental leadership and employees’ organizational citizenship behavior. *Int. J. Hospital. Manag.* 87:102375 10.1016/j.ijhm.2019.102375

[B31] Kura K. M. (2016). Linking environmentally specific transformational leadership and environmental concern to green behaviour at work. *Glob. Bus. Rev.* 17 1S–14S. 10.1177/0972150916631069

[B32] Lavergne K. J. Sharp E. C. Pelletier L. G. Holtby A. (2010). The role of perceived government style in the facilitation of self-determined and non self-determined motivation for pro-environmental behavior. *J. Environ. Psychol.* 30 169–177. 10.1016/j.jenvp.2009.11.002

[B33] Liu D. Chen X. P. Yao X. (2011). From autonomy to creativity: a multilevel investigation of the mediating role of harmonious passion. *J. Appl. Psychol.* 96 294–309. 10.1037/a0021294 21058804

[B34] Liu W. Tian J. Chen L. Lu W. Gao Y. (2016). Environmental performance analysis of eco-industrial parks in china: a data envelopment analysis approach. *J. Indus. Ecol.* 19 1070–1081. 10.1111/jiec.12233

[B35] Lu H. Liu X. Chen H. Long R. Yue T. (2017). Who contributed to “corporation green” in China? A view of public- and private-sphere pro-environmental behavior among employees. *Resour. Conserv. Recycl.* 120 166–175. 10.1016/j.resconrec.2016.12.008

[B36] Molina-Azorín J. F. Tarí J. J. Pereira-Moliner J. López-Gamero M. D. Pertusa-Ortega E. M. (2015). The effects of quality and environmental management on competitive advantage: a mixed methods study in the hotel industry. *Tour. Manag.* 50 41–54. 10.1016/j.tourman.2015.01.008

[B37] Morton K. L. Barling J. Rhodes R. E. Masse L. C. Zumbo B. D. Beauchamp M. R. (2011). The application of transformational leadership theory to parenting: questionnaire development and implications for adolescent self-regulatory efficacy and life satisfaction. *J. Sport Exerc. Psychol.* 33 688–709. 10.1123/jsep.33.5.688 21984642

[B38] Nohe C. Hertel G. (2017). Transformational leadership and organizational citizenship behavior: a meta-analytic test of underlying mechanisms. *Front. Psychol.* 8:1364. 10.3389/fpsyg.2017.01364 28848478 PMC5554340

[B39] Norton T. A. Parker S. L. Zacher H. Ashkanasy N. M. (2015). Employee green behavior A theoretical framework, multilevel review, and future research agenda. *Organ. Environ.* 28 103–125. 10.14264/uql.2015.308

[B40] Norton T. A. Zacher H. Ashkanasy N. M. (2012). On the importance of pro-environmental organizational climate for employee green behavior. *Indus. Organ. Psychol.* 5 497–500. 10.1111/j.1754-9434.2012.01487.x

[B41] Norton T. A. Zacher H. Parker S. L. Ashkanasy N. M. (2017). Bridging the gap between green behavioral intentions and employee green behavior: the role of green psychological climate. *J. Organ. Behav.* 38 996–1015. 10.1002/job.2178

[B42] Osbaldiston R. Sheldon K. M. (2003). Promoting internalized motivation for environmentally responsible behavior: a prospective study of environmental goals. *J. Environ. Psychol.* 23 349–357. 10.1016/S0272-4944(03)00035-5

[B43] Pelletier L. G. Dion S. Tuson K. Green-Demers I. (2010). Why do people fail to adopt environmental protective behaviors? Toward a taxonomy of environmental amotivation. *J. Appl. Soc. Psychol.* 29 2481–2504. 10.1111/j.1559-1816.1999.tb00122.x

[B44] Perrewé P. L. Hochwarter W. A. Ferris G. R. Mcallister C. P. Harris J. N. (2013). Developing a passion for work passion: future directions on an emerging construct. *J. Organ. Behav.* 35 145–150. 10.1002/job.1902

[B45] Perttula K. H. Cardon M. S. (2011). “Passion,” in *The Oxford Handbook of Positive Organizational Scholarship*, eds Spreitzer G. M. Cameron K. S. (New York, NY: Oxford University Press), 190–200.

[B46] Podsakoff P. M. MacKenzie S. B. Lee J. Y. Podsakoff N. P. (2003). Common method biases in behavioral research: a critical review of the literature and recommended remedies. *J. Appl. Psychol.* 88 879–903. 10.1037/0021-9010.88.5.879 14516251

[B47] Podsakoff P. M. MacKenzie S. B. Podsakoff N. P. (2012). Sources of method bias in social science research and recommendations on how to control it. *Annu. Rev. Psychol.* 63 539–569. 10.1146/annurev-psych-120710-100452 21838546

[B48] Podsakoff P. M. Organ D. W. (1986). Self-reports in organizational research: problems and prospects. *J. Manag.* 12 531–544. 10.1177/014920638601200408

[B49] Preacher K. J. Hayes A. F. (2008). Asymptotic and resampling strategies for assessing and comparing indirect effects in multiple mediator models. *Behav. Res. Methods* 40 879–891. 10.3758/BRM.40.3.879 18697684

[B50] Raineri N. Paillé P. (2016). Linking corporate policy and supervisory support with environmental citizenship behaviors: the role of employee environmental beliefs and commitment. *J. Bus. Ethics* 137 129–148. 10.1007/s10551-015-2548-x

[B51] Robertson J. L. (2018). The nature, measurement and nomological network of environmentally specific transformational leadership. *J. Bus. Ethics* 151 961–975. 10.1007/s10551-017-3569-4

[B52] Robertson J. L. Barling J. (2013). Greening organizations through leaders’ influence on employees’ pro-environmental behaviors. *J. Organ. Behav.* 34 176–194. 10.1002/job.1820

[B53] Robertson J. L. Carleton E. (2018). Uncovering how and when environmental leadership affects employees’ voluntary pro-environmental behavior. *J. Leadersh. Organ. Stud.* 25 197–210. 10.1177/1548051817738940

[B54] Schmitt A. Den Hartog D. N. Belschak F. D. (2016). Transformational leadership and proactive work behaviour: a moderated mediation model including work engagement and job strain. *J. Occup. Organ. Psychol.* 89 588–610. 10.1111/joop.12143

[B55] Simmering M. J. Fuller C. M. Richardson H. A. Ocal Y. Atinc G. M. (2015). Marker variable choice, reporting, and interpretation in the detection of common method variance: a review and demonstration. *Organ. Res. Methods* 18 473–511. 10.1177/1094428114560023

[B56] Steg L. Bolderdijk J. W. Keizer K. Perlaviciute G. (2014). An integrated framework for encouraging pro-environmental behaviour: the role of values, situational factors and goals. *J. Environ. Psychol.* 38 104–115. 10.1016/j.jenvp.2014.01.002

[B57] Thompson G. Vecchio R. P. (2009). Situational leadership theory: a test of three versions. *Leadersh. Q.* 20 837–848. 10.1016/j.leaqua.2009.06.014

[B58] Tuan L. T. (2019a). Catalyzing employee OCBE in tour companies: charismatic leadership, organizational justice, and pro-environmental behaviors. *J. Hospital. Tour. Res.* 43 682–711. 10.1177/1096348018817582

[B59] Tuan L. T. (2019b). Effects of environmentally-specific servant leadership on green performance via green climate and green crafting. *Asia Pacific J. Manag.* 10.1007/s10490-019-09687-9 [Epub ahead of print].

[B60] Turaga R. M. R. Howarth R. B. Borsuk M. E. (2010). Pro-environmental behavior: rational choice meets moral motivation. *Ann. N. Y. Acad. Sci.* 1185 211–224. 10.1111/j.1749-6632.2009.05163.x 20146771

[B61] Vallerand R. J. Blanchard C. Mageau G. A. Koestner R. Ratelle C. Leonard M. (2003). Les passions de l’ame: on obsessive and harmonious passion. *J. Pers. Soc. Psychol.* 85 756–767. 10.1037/0022-3514.85.4.756 14561128

[B62] Walumbwa F. O. Avolio B. J. Zhu W. (2008). How transformational leadership weaves its influence on individual job performance: the role of identification and efficacy beliefs. *Pers. Psychol.* 61 793–825. 10.1111/j.1744-6570.2008.00131.x

[B63] Wang X. Zhou K. Liu W. (2018). Value congruence: a study of green transformational leadership and employee green behavior. *Front. Psychol.* 9:1946. 10.3389/fpsyg.2018.01946 30356727 PMC6189445

[B64] Weiss H. M. Cropanzano R. (1996). Affective events theory: a theoretical discussion of the structure, causes and consequences of affective experiences at work. *Res. Organ. Behav.* 18 1–74. 10.1177/030639689603700317

[B65] Wesselink R. Blok V. Ringersma J. (2017). Pro-environmental behaviour in the workplace and the role of managers and organisation. *J. Clean. Product.* 168 1679–1687. 10.1016/j.jclepro.2017.08.214

[B66] Williams L. J. Hartman N. Cavazotte F. (2010). Method variance and marker variables: a review and comprehensive CFA marker technique. *Organ. Res. Methods* 13 477–514. 10.1177/1094428110366036

[B67] Xie S. Zhang W. (2012). The relationships between transformational leadership, LMX, and employee innovative behavior. *J. Appl. Bus. Econ.* 13 87–96.

[B68] Yuriev A. Boiral O. Francoeur V. Paillé P. (2018). Overcoming the barriers to pro-environmental behaviors in the workplace: a systematic review. *J. Clean. Product.* 182 379–394. 10.1016/j.jclepro.2018.02.041

[B69] Zerbe W. J. Härtel C. E. J. Ashkanasy N. M. (2008). The role of emotions in driving workplace pro-environmental behaviors. *Res. Emot. Organ.* 4 83–107. 10.1016/S1746-9791(08)04004-2

